# In silico analyzing the molecular interactions of plant-derived inhibitors against E6AP, p53, and c-Myc binding sites of HPV type 16 E6 oncoprotein

**DOI:** 10.22099/mbrc.2020.36522.1483

**Published:** 2020-06

**Authors:** Farzan Nabati, Mohammad Moradi, Hassan Mohabatkar

**Affiliations:** Department of Biotechnology, Faculty of Biological Science and Technology, University of Isfahan, Isfahan, Iran

**Keywords:** HPV16 E6, Natural inhibitors, Molecular docking, E6AP, p53, Myc

## Abstract

Human papillomaviruses (HPV) are a group of strong human carcinogen viruses considered to be the fourth leading cause of mortality among women in the world. HPV is the most important cause of cervical cancer, which is the second most common cancer in women living in low and middle-income countries. To date, there is no effective cure for an ongoing HPV infection; therefore, it is required to investigate anticancer drugs against this life-threatening infection. In this study, we collected more than 100 plant-derived compounds with anti-cancer and antiviral potentials from a variety of papers. Smile formats of these compounds (ligand), were harvested from PubChem database and examined based on the absorption, distribution, metabolism, excretion, and toxicity properties by programs such as Swiss ADME, admetSAR, and pkCSM. Twenty compounds, which were likely to be the HPV16E6 inhibitor, were selected for docking calculations. We examined these natural inhibitors against the HPV16 E6 oncogenic protein. Eventually, three of these compounds were used as the most potent inhibitors (Ginkgetin (*peculiarly*), Hypericin and Apigetrin) were probably used as the possible source of cancer treatment caused by E6 oncoprotein. In this research, we conducted the docking calculations by Autodock 4.2.6 software. Docking analysis showed the interaction of these plant-originated inhibitors with E6AP, p53, and Myc binding sites on the E6 oncoprotein which support the normal function of E6AP, p53, and Myc.

## INTRODUCTION

Over 100 types of cancers affect humans, which is the second-leading cause of mortality worldwide and responsible for 8.8 million deaths in 2015. Expected by 2020, this cancer causes up to 10 million deaths (World Health Organization report) [1]. Some researchers believe that most cancers (about 90-95%) are due to genetic mutations caused by environmental factors and lifestyle, and the remaining 5-10% are due to inherited genetics [2, 3]. Worldwide, approximately 18 percent of mortality from cancer are related to infectious agents [4]. Inhibiting the activity of major transcription factors has shown to be a high potential approach for cancer treatment *[*5*, *6*].*

Papillomaviruses are a group of the most commonly found viruses that could cause cancer in humans. It has been reported that some strains of human papillomavirus (HPV) cause cervical cancer [7]. Over 200 different HPV types have been identified to infect human and are classified into two different groups: high risk and low risk [8]. High-risk genotypes, including HPV16, 18, 45 and 33 (63, 11, 6, and 4%, respectively) are more commonly associated with cervical cancers, whereas low-risk types such as HPV6 and 11, typically cause genital warts [9, 10]. 

High-risk types of HPV are the causative agents of over 99.7% of cervical cancers [11]. Human papillomavirus type 16 is known as a major causative factor in the development of cervical carcinomas [12]. Estimates of the global cancer burden indicate that cervical cancer is increasingly prevalent in low- and middle-income countries [13-17].

HPVs are a group of small non-enveloped, icosahedral tumor viruses that possess a circular double-stranded DNA genome with a size of ~55 nm in diameter that infects cutaneous or mucosal epithelial cells, causing papillomata or warts on the skin, genital tissues, and the upper respiratory tract [18]. The HPV genome consists of approximately 8000 base pairs [19]. Functionally, the genome of the HPV is divided into three distinct regions. The first long control region (LCR or URR (upstream regulatory region)) is responsible for the regulation and control of viral DNA replication and transcription. The early region contains six ORFs (open reading frames) and encoding non-structural viral regulatory proteins and viral replication (E1, E2, E4), three of which, E5, E6, and E7, are oncogenic. The late genes region encodes structural proteins involved in the formation of the viral capsid proteins L1 and L2, and varies between different HPV types, which is necessary for virion transmission and spread [20, 21].

The E6 is one of the two oncoproteins expressed in the oncogenic human papillomavirus types 16 and 18, which plays a major role in malignant transformation, carcinogenicity, and immortality [22-24]. The E6 is an oncoprotein composed of polypeptides made up of ~150 amino acids long with a molecular weight between 16-18 kDa and two Cys-X-X-Cys motifs that allow the formation of two zinc fingers [25, 26]. E6 oncoproteins are expressed as a full-length (16-18 kDa) protein as well as its splice isoform E6* (7 kDa), in the host cells [27]. It has been shown that the expression of the HPV16 E6* isoform increases oxidative stress and induces oxidative DNA damage in the host cells [28, 29].

 E6 oncoprotein interacts with the binding of several cellular proteins via two known binding motifs, namely, the acidic LXXLL motif (e.g., E6-AP, c-Myc, p53), PDZ domain (e.g., hScrib, hDlg, MAGI-1 (-2, -3), MUPP1, PATJ, and PTPN3) and unknown E6 binding motif (e.g., FADD, Gps2, hADA3, and Procaspase) [30-32]. Mainly, this oncoprotein by forming complexes with cellular proteins, stimulates the destruction of many host cell's key regulatory proteins such as E6AP, p53, and c-Myc, which could result in cancer.

P53 is the most important human protein, which is involved in cell cycle regulation, apoptosis, as well as playing a significant role in maintaining genetic stability conditions. When the human cell's genome is damaged for any cause, and these damaged cells do not enter the cell proliferation cycle, the rate of p53 increases rapidly and induces apoptosis of the damaged cells [21]. High-risk E6 oncoproteins mediate the ubiquitination and cellular ubiquitin ligase protein called E6AP (E6-Associated Protein) that catalyzes the degradation of p53 through the formation of a heterotrimeric complex (E6/E6AP/p53). Direct binding between E6, p53, and E6AP forms this complex. [33]. When the content of p53 decreases, after degradation by E6 oncoprotein of the host cells, they cannot ordinarily repair DNA damages, which ultimately leads to malignancy.

Myc (transcription factor) is one of the E6 partners that regulates the expression of up to 10-15% of the cellular genes controlling metabolic processes, post-translational modifications, macromolecular synthesis, cellular proliferation, and apoptosis [34]. hTERT (human telomerase reverse transcriptase ), the catalytic subunit of telomerase, is one of the Myc targets [35]. Telomerase is a complex of ribonucleoprotein enzymes, consisting of two core subunits, called template RNA subunit (human telomerase RNA (hTR)) and a catalytic protein subunit (hTERT), that extends telomeric DNA. The telomere DNA is a repetitive and nucleoprotein complex located at the ends of eukaryotic chromosomes with approximately 5,000 to 15,000 nucleotides length in humans. Without the telomerase activity, the linear chromosomes of cellular DNA serially are shortened with each cell cycle and get divided by 100 to 200 nucleotides, then cells enter the mortality stage [35, 36]. New studies have shown that cells infected with E6 protein hr-HPV use c-Myc and directly interact with hTERT, leading to increased phosphorylation of RNA Pol II and induced epigenetic histone modifications of the hTERT promoter, therefore it triggers the activation of the hTERT promoter, which increases the telomerase activity [37]. Increased expression of hTERT in the most human cancers, including HPV-positive cervical cancer, prevents the shortening of telomere length, leading to cell immortalization.

In this study, twenty different natural compounds from several plant sources for molecular docking calculations were collected (Supplement file, Table S1), and we investigated the molecular interaction of the HPV16 E6 protein with plant-originated inhibitors around E6AP, p53, and Myc binding site respectively.

## MATERIALS AND METHODS

**Computer programs: **Bioinformatics programs such as Chimera 1.12 [38], Molegro Virtual Docker v 6.0 (MVD), YASARA Energy Minimization, Hex software, LigPlot^+^ (v.1.4.5) were used [39] and Molecular docking calculation was performed by AutoDock tools 1.5.6 and MGL tools 1.5.6 packages [40,41]. Furthermore, online resources such as Herbmed, Sciencedirect, Pubmed, Google Scholar, Pubchem database [42], Pkcsm [43], Swissadme [44], admetSAR [45], Protein Data Bank, NCBI, Phyre 2 server [46], ProSAWeb [47], Verify-3D [48], ProQ [49], Procheck [50], and ERRAT serve [51] and Ramachandran plot [52] were used for data collection.

**Docking Procedure**: More than 100 natural compounds representing antiviral and anticancer activity were collected from the literature; retrieved from http://www.herbmed.org, Pubmed, Google Scholar, ScienceDirect. All the natural compounds reported were examined based on Absorption (Log s), high gastrointestinal absorption (GI), distribution (Log D), metabolism (CYP3A4, CYP2D6, CYP2C9, CYP2C19, and CYP1A2), excretion, and toxicity (Hepatotoxicity, hERG (I, II) inhibitor, AMES Toxicity, Carcinogens, and Lipinski [53], Lipophilicity (Log p), polar surface area (Å²) by online servers such as Swiss ADME, admetSAR, and pkCSM. 

The compounds were not supposed to inhibit cytochromes (CYP3A4, CYP2D6, CYP2C9, CYP2C19, and CYP1A2) and have high water solubility) Log s<6), Log p be in the range of - 0.7 < log p < 5, and TPSA be ranging 20 < TPSA < 130 Å². After the filtering operation by online servers, 20 of the 100 natural compounds were selected for molecular docking calculations (Table S1). Chemical structures of twenty natural compounds were retrieved from the PubChem database. The structures were downloaded in smiles format and converted to PDB using Chimera 1.12 then PDB format's structures were minimized by chimera 1.12 and were saved in PDB files. PDB file was submitted to ADT (AutoDock Tools is a set of commands implemented within the Python Molecular Viewer (PMV)) for PDBQT file preparation. Also, docking calculations were carried out on ligands by ADT (1.5.6), AutoDock4.2 program operated Docking calculation. Three-dimensional structure of E6 (PDB ID: 4GIZ) was retrieved from the PDB database. Initially, extra chains (A, B, and D), water molecules, and ligands of PDB file (4GIZ) retrieved from Protein Data Bank were removed by MVD. Finally, the C chain (4GIZ) was submitted to the MVD for optimizing/repair, then the PDB file was evaluated and passed to Autodock Tools (ADT ver.1.5.6) for PDBQT File preparation. Therefore, non-standard residues were removed. Only polar hydrogens were maintained, and Gasteiger charges were computed for protein atoms, Kollman charges were also assigned by ADT, The PDB file was prepared for Docking with Autodock 4.2.6. Initially, for the studies of molecular Docking simulations E6 with Myc, we predicted Myc 3D protein structure by online servers. Since the three-dimensional structure (3D) of Myc retrieved from the protein data bank, (ID: 1NKP) had a problem, owing to four amino acids (30, 31, 32, and 33) to be in the error region. 

**Figure 1:**
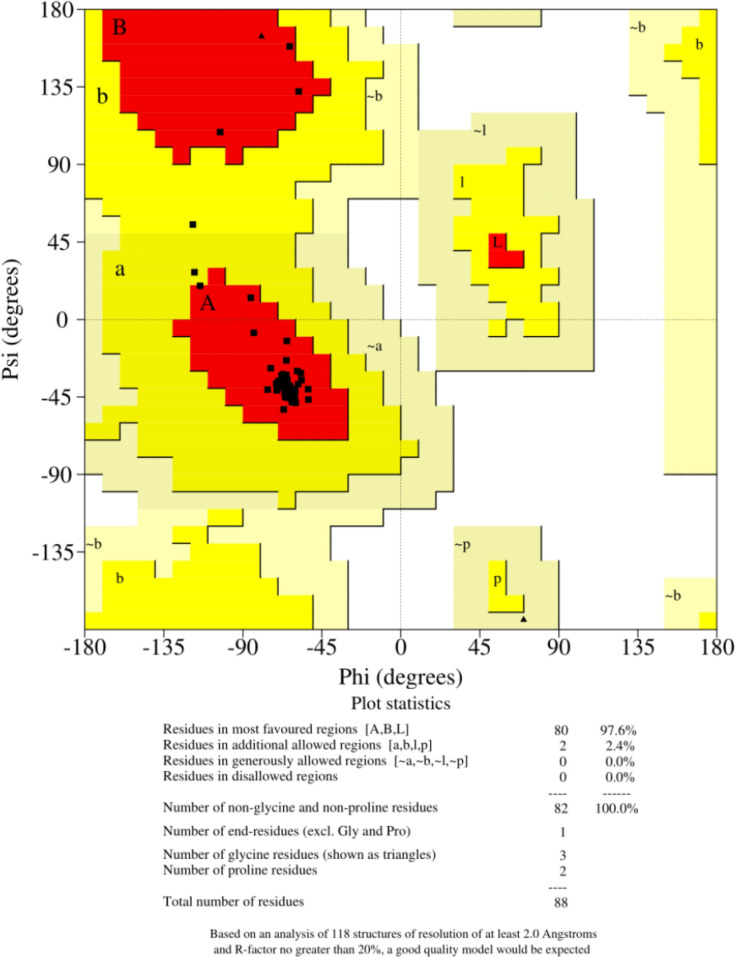
Validation results for the modeled c-Myc (Ramachandran plot of model)

The Myc Ref.sequence (NP_002458.2) was harvested from NCBI. For the modeling of the 3D structure of the Myc protein, Phyre 2 server was used, and the result of the Phyre 2 server query was as following: coverage of the target-template alignment was 99.61% confidence with 98% identity. Structural refinement and energy minimization of the predicted model was carried out using YASARA Energy Minimization program. The total energy minimization for the refined structure obtained from the YASARA program was -66671.8 kJ/mol (score, 2.40), whereas, before energy minimization, it was -50950 kJ/mol (score, 0.52). The refined model reliability was assessed through Procheck, ProSA-Web, ProQ and, Verify-3D. Finally, Myc 3D structure was refined for further confirmation by the ERRAT online server. The Ramachandran plot for the Myc 3D structure was created by Phyre 2 server was as following: the 97.6% residues were in the favorable regions, 2.4% residues were in the allowed region, and no amino acids were found in the outlier region (Fig. 1).

**Determining the binding sites of E6 to E6AP, p53, and Myc** **: **We explained the atomic interaction between plant-originated ligands and high-risk HPV16 E6 oncogenic protein. These natural plant compounds might prevent and disable E6 oncoprotein from interacting with E6AP, p53, and Myc proteins. However, when E6 interacts with host E6AP, p53 and Myc protein, it plays important roles in the induction of cell immortality All residues were involved in the binding of E6 to E6AP consist of R10, K11, V31, Y32, L50, C51, V53, R55, V62, L67, Y70, S74, R77, H78, L100, R102, Q107, R129, and R131. Residue interface in hydrophobic interactions such as: V31, Y32, L50, C51, V53, V62, L67, Y70, L100, R102, Q107, R131 and residue interface in polar interactions involve side-chain groups such as R10, R55, S74, R77, H78, R129, R131 and residue interface in polar interactions involve backbone groups (main chain) such as K11, C51, R102, and R131 [54]. Autodock calculated the region of interest for docking runs; the grid map was formed based on polar residues in the binding site of receptor (E6 oncoprotein) proteins to E6AP for the docking process. The dimension of the grid map was calculated based on polar residue in the binding site using AutoGrid (part of the AutoDock package) and drawn. The dimension of the grid map was given as x-dimension = 56, number of points in y-dimension = 50 and number of points in z-dimension = 86 points in a grid space of 0.375 Å, centers of grid box: X = 2.851; Y = 50.522; Z = 27.126. All residues were involved in the binding of E6 to p53 including Q6, E7, R8, R10, Q14, E18, Y43, D44, F47, D49, L100, and P112. Residue interface in hydrophobic or non-polar interactions were including Q6, E7, R8, R10, Q14, E18, Y43, D44, F47, D49, L100, and P112. Residue interface in polar interactions involve side-chain groups were including E7, R10, Q14, E18, D44, D49 and residue interface in polar interactions involve backbone groups (main chain) were including Q6, E7, R8, R10, and E18 [55].

AutoGrid was used to create a grid map; the grid map was formed based on polar residues in the binding site of receptor (E6 oncoprotein) proteins to p53 for docking process. The dimension of the grid map was number of points in x-dimension = 64, number of points in y-dimension = 86 and number of points in z-dimension = 106 points in a grid space of 0.375 Å, centers of the grid box: X = 6.00; Y = 61.00; and Z= 23.700. 

HEX 8.0.0 v 2013 software was used to predict the E6 interaction to Myc. It was indicated that E6 is interfacing with Myc interact through domain Z finger-1 (N-Terminal). Residues around such as F2, P5, R8, and Y54 interacted with human Myc protein. The region of the grid map was used by Autodock in docking runs as defined and calculated by AutoGrid. The grid map was formed based on all the amino acids of E6 oncoprotein that are involved in the Myc binding site was formed. The dimension of the grid map was number of points in x-dimension =50, number of points in y-dimension =50 and number of points in z-dimension =50 points in a grid space of 0.375 Å, centers of the grid box: X = 3.200; Y = 55.00; and Z = 13.00.

**Docking Analysis of E6** **oncoprotein** **with plant-derived inhibitors: **Molecular docking operation was performed in various conformations on all the plant-originated inhibitors with different binding energy. Finally, the compounds with the lowest energy conformation of the E6 were selected. Upon docking, the binding energies of E6 oncoprotein with plant-originated ligands were obtained; the complete detail of interactions are provided in Table 1. 

The PDB files (receptor and ligands) prepared for docking operation were submitted to ADT for PDBQT file preparation. Docking calculations were carried out on receptors such as delete water molecules, non-standard residues were removed, only polar hydrogen was maintained, and Gasteiger charges were computed for protein atoms, Kollman charges were also assigned. As well as Docking, calculations on ligand such as computing Gasteiger charges, merged non-polar hydrogens, found aromatic carbons, detected rotatable bonds and set TORSDOF carried out by ADT automatically. After docking calculations, PDBQT files were saved for docking computations with the AutoDock4.2 program. The grid maps representing the receptor proteins in the docking process were calculated (x, y, and z dimension) using AutoGrid (see above). The Lamarckian genetic algorithm (LGA) was applied to the interaction pattern between the receptor and selected natural metabolite inhibitors (ligand), with 100 genetic algorithms (GA) runs. Other settings for the Docking process was considered a default, such as the population size of 150 was determined for each 25 × 105 energy evaluations, 27,000 maximum number of generations, 0.02 rate of gene mutation, and a crossover rate of 0.8 were used for the LGA.

**Table 1:** The result of docking analysis between ligands isolated from plant-originated compounds with the binding sites of an E6 Oncoprotein to E6AP, P53, and Myc

**Row**	**Compound name**	**Docking** **Score** ^i^ **(kcal/mol)**		**Inhibition constant** **(μ** **M)**	**Number of hydrogen bonds**	**Residues** **involved in hydrogen bonding**
**1**	Apigenin	E6AP	-5.8	57.64	2	Leu67, Cys51
		P53	-6.37	21.37	2	Leu67, Cys51
		Myc	-5.57	98.25	2	Arg8, Arg55
**2**	Apigetrin	E6AP	-6.37	21.27	4	2^i^ Cys51, Arg55, Pro5
		P53	-7.61	2.63	6	Pro5, 3 Cys51, 2 Arg55
		Myc	-6.13	32.2	3	2 Phe2, Asn58
**3**	Aplysin	E6AP	-6.01	39.59	--------	**---------------------**
		P53	-6.26	32.23	1	Trp132
		Myc	-5.53	89.11	1	Gln3
**4**	Curcumin	E6AP	-5.78	58.09	2	2 Cys51
		P53	-6.21	28.24	2	2 Cys51
		Myc	-6.49	17.51	3	Tyr54, Asp56, Gly57
**5**	Epicatechin	E6AP	-5.4	109.54	5	2 Pro5, Ile52, Arg55, Tyr54
		P53	-5.95	43.64	5	2 Pro5, Tyr54, Arg55, Ile52
		Myc	-5.69	67.42	4	Tyr54, Arg55, Arg8, Pro5
**6**	Eurycomanone	E6AP	-6.13	32.25	3	2 Cys51, Tyr32
		P53	-6.81	10.25	3	2 Cys51, Tyr32
		Myc	-5.45	101,28	3	Pro5, Arg10, Arg8
**7**	Genistein	E6AP	-6.68	12.74	3	Ser71, Cys51, Tyr32
		P53	-6.66	13.03	2	Ser71, Tyr32
		Myc	-6.26	25.74	5	3 Arg8, Arg55, Tyr54
**8**	Gingerol	E6AP	-4.58	436.16	2	2Cys51
		P53	-5.03	205.08	4	2Arg8, Arg55, Tyr54
		Myc	-4.46	534.89	3	Arg8, Arg10, Arg55
**9**	Ginkgetin	E6AP	-8.45	0.642	5	2 Arg55, Cys51, Val 53, Tyr 60
		P53	-8.46	0.632	5	2 Arg55, Cys51, Val 53, Tyr 60
		Myc	-7.22	5.11	4	Pro5, 3Arg8
**10**	Hypericin	E6AP	-7.15	5.7	5	2 Gln6, Tyr54, Ile52, Pro5
		P53	-7.24	4.97	5	Ile52, Tyr54, 2 Gln6, Pro5
		Myc	-6.67	12.99	3	Ile52, Pro5, Arg55
**11**	Isonoruon	E6AP	-5.78	58.08	1	Cys51
		P53	-5.81	55.3	1	Cys51
		Myc	-5.65	72.48	1	Arg8
**12**	Klaineanone	E6AP	-6.41	20.12	3	3 Cys51
		P53	-6.8	10.44	3	Ile101, Lys115, Ser97
		Myc	-6.29	24.52	3	Ile52, Arg10, Tyr54
**13**	L-Ectone	E6AP	-5.12	177.32	2	2 Cys51
		P53	-5.28	134.39	1	Leu110
		Myc	-5.05	198.99	1	Arg8
**14**	Oleocanthal	E6AP	-3.79	1680	2	Leu67, Cys51
		P53	-5.6	78.55	5	Tyr54, 2 Arg8, Gln6, Arg55
		Myc	-5.43	105.11	4	Gln6, Arg8, Arg10, Tyr54
**15**	Oleuropein	E6AP	-4.36	640.94	4	2 His78, Ser74, Arg131
		P53	-5.02	208.38	3	Tyr70, Arg13, Cys51
		Myc	-5.05	198.09	8	2 Gln6, 2 Arg8, 2 Arg55, Pro5, Arg10
**16**	Piceatannol	E6AP	-5.3	129.35	3	Ser71, 2 Cys51
		P53	-6.1	33.96	5	Lys115, Arg47, 2 Leu99, Ile101
		Myc	-5.64	73.41	5	2 Glu18, 2 Asp4, Gln7
**17**	Pterostilbene	E6AP	-5.74	61.921	1	Cys51
		P53	-5.64	73.63	1	Cys51
		Myc	-5.08	188.6	2	2 Asp4
**18**	Quercetin	E6AP	-4.33	674.16	5	Ser71, Tyr32, Ile104, Ser74, Arg129
		P53	-6.39	20.75	7	2 Leu99, Arg47, Lys108, 2 Asp49, Ile101
		Myc	-5.29	132.96	6	2 Asn58, 2 Glu18, Gln3, Gly57
**19**	Silibinin	E6AP	-6.89	8.88	4	2 Cys51, Arg8, Pro5
		P53	-7	7.38	5	Gln14, 2 Arg8, Arg10, Gln6
		Myc	-6.01	39.06	4	2 Arg55, Arg8, Pro5
**20**	Tyrosol	E6AP	-4.33	672.1	2	Tyr32, Cys51
		P53	-4.42	573.57	3	2 Asp4, Glu18
		Myc	-4.63	402.47	2	2 Asp4
**+**	Paclitaxel (Positive control)	E6AP	-5.99	40.87	2	Cys51, Ser74
		P53	-7.59	2.73	4	Tyr32, Ile52, Arg8, Arg55
		Myc	-5.4	110.55	4	2 Arg10, Arg55, Arg8

(a) Indicates interactions between the binding sites of E6 to E6AP, P53, and Myc with natural inhibitors. (b)The number before the amino acids indicates the number of hydrogen bonds that amino acids of E6 establish with the ligand.

## RESULTS

Docking analysis showed that all 20 natural ligands bind to three binding sites on HPV E6 oncoproteins that can help the restoration of the normal functioning of tumor suppressor proteins, and the lowest binding energy conformation was analyzed and presented in Table 1. It was showed that interaction of all 20 natural ligands with HPV E6 oncoproteins, and among them, Ginkgetin (GK) (especially), Hypericin, and Apigetrin have effectively inhibited three binding sites on HPV E6 oncoproteins with minimum binding energy.

**Figure 2:**
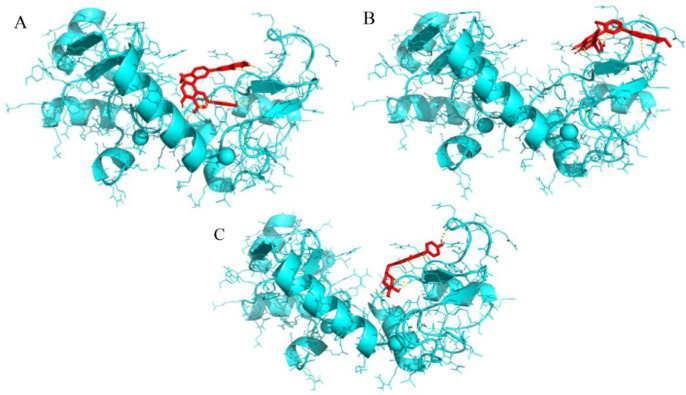
3D Molecular Interactions between E6 binding sites to E6AP (A), p53 (B), and c-Myc (C) with Ginkgetin

Among the three natural ligands (GK, Hypericin, and Apigetrin) selected, GK most effectively with the lowest binding energy interacted with all three binding sites E6AP, p53, and myc on E6 oncoproteins. (The three-dimensional structure of these interactions are shown in Figure 2 (A, B, and C respectively). GK showed the lowest binding energy (-8.45 kcal/mol) with the E6AP binding site on HPV-16 E6 protein and inhibition constant (0.642 μM) for the protein-ligand complex.

**Figure 3:**
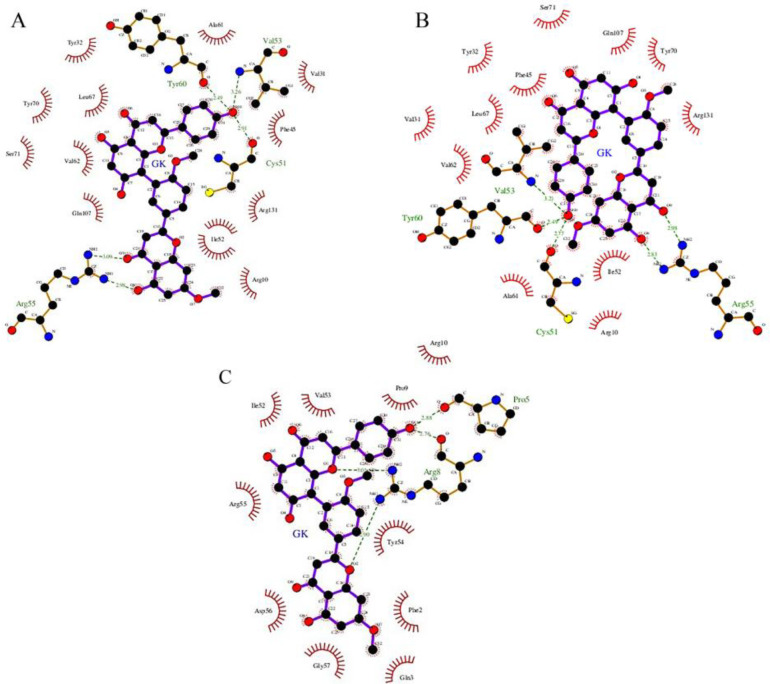
2D Molecular Interactions between E6 binding sites to E6AP (A), p53 (B), and c-Myc (C) with Ginkgetin. The hydrogen bonds are shown as dash lines and hydrophobic contacts are indicated with half-moon

The four amino acid residues of HPV-16 E6, (i.e., Arg 55, Cys 51, Val 53, and Tyr 60) were observed to form five hydrogen bonds with GK during protein, ligand interactions (Fig. 3A). Similarly, the binding energy of GK with the p53 binding site on HPV-16 E6 protein was showed to be a minimum binding energy of -8.46 kcal/mol with an inhibition constant of 0.632 μM. GK formed five hydrogen bonds with four amino acid residues (i.e.; Arg 55, Cys 51, Val 53, and Tyr 60) from the HPV-16E6 protein (Fig. 3B). In the case of the binding energy of GK with Myc binding site on HPV-16, E6 protein interacted with two amino acid residues from the receptor (i.e., Pro 5, and Arg 8) by forming four hydrogen bonds (Fig. 3C), the binding energy of the interaction was -7.22 kcal/mol, and the inhibition constant was 5.11 μM (Table 1).

The study showed that GK is an effective inhibitor of the binding sites available on the E6. This computational approach demonstrates the effectiveness of GK as an anticancer agent that needs to be explored further until we learn more about how to use GK or others natural products for designing novel drugs against cervical cancer.

## DISCUSSION

Approximately 1.6 million of the 2 million new cases of cancer each year are caused by infection with carcinogenic viruses. This issue has caused to focuses on new treatments for these viruses and their mechanisms of action [1]. Carcinogenic viruses, especially oncogenes produced by them with several different molecular mechanisms modify normal cells to cancer cells. Viral oncoproteins like E6, form complexes with cellular proteins, causes broad biological changes including; regulating cellular pathways, preventing shortening of the telomeres, immortalization, host cell differentiation, regulating growth factors, degradation and inactivation tumor suppressor, interference with DNA repair efficiency, and apoptosis**,** promote cell transformation, increase of the host genetic and epigenetic alterations etc. In addition, carcinogenic viruses cause genetic disorders by entering their genome into host cell chromosomes [30,56,57]. Although several viruses can cause various tumors in animals, only seven kinds of them are linked with cancer in humans [1, 58].

Cervical cancer is one of the most dangerous and deadly cancers in women caused by HPV. There are several options for the treatment of early-stage cervical cancer such as surgery, non-specific chemotherapy, radiation therapy, laser therapy, hormonal therapy, targeted therapy, and immunotherapy, but there is no effective cure for an ongoing HPV infection. Herbal extracts are one of the therapeutic areas for cervical cancer; many researchers have studied the effect of plant metabolites on cervical cancer treatment. Researchers have demonstrated that curcumin, epigallocatechin-3-gallate (EGCG), jaceosidin, resveratrol, indole-3- carbinol, withaferin A, artemisinin, ursolic acid, ferulic acid, berberin, resveratrol, gingerol, and silymarin compounds are possible effective agents for cancer treatment [59,60]. Traditionally, different plant-originated compounds have been identified and tested as promising resources against cancer caused by HPV.

 Advancements in computational biology and bioinformatics are useful to the investigation of novel inhibitors from herbal medicines against cancers like cervical cancer. Computer-aided docking is a valuable tool for gaining an understanding of the binding interactions between a ligand (small molecule) and its receptor (macromolecule) and has emerged as a reliable, cost-effective, time-saving and fast technique for the discovery of novel drugs [61]. Molecular docking provides the following three main goals: predicting the ligand binding site to the receptor, virtual screening, and binding affinity calculation [62]. Molecular docking studies further help to understand the various interactions between the small molecules and particular receptor targets binding sites and is used as a standard computational tool to design novel potent inhibitors.

 The high-risk HPV type 16 has oncoprotein (E6), that need to stay blocked for various reasons, including the fact that E6 is able to be inactivated tumor suppressor proteins (p53) by the E6AP human protein, then causes p53 degradation by the proteasome pathway. In addition, E6/ Myc interactions cause to induce the transactivation hTERT promoter and the increase of telomerase activity, finally leading to tumor cells immortalization. Therefore, E6 oncoprotein is a very important goal in designing new inhibitors against cervical cancer. 

GK is a natural bioflavonoid isolated from leaves of Ginkgo biloba (Ginkgoaceae). Ginkgo biloba (GB) is originated in Asia, particularly in southeast China, where more than 4000 years it has been used as traditional herbal medicine. Extracts from GB leaves contain various glycosides and terpenoids and have been found to exert different pharmacological actions. Among several compounds, isolated from GB leaves, GK has shown various biological functions including the potent anti-inflammatory and anti-viral, antifungal, neuroprotective, anti-influenza, anti-arthritic and antitumor activities [63]. Researchers have reported that GK inhibits the growth of prostate and breast cancer cells [64,65], and inhibits the proliferation of human leukemia cells [66]. Also, GK induces autophagy and apoptosis in cancer cells [67, 68]. In the present study, bioinformatics simulation showed the anticancer properties of GK against cervical cancer. Generally, GK by blocking the binding site of E6 oncoprotein to E6AP, p53, and Myc, will support the normal function of these intracellular proteins.
